# Sporicidal activity of ceragenin CSA-13 against *Bacillus subtilis*

**DOI:** 10.1038/srep44452

**Published:** 2017-03-15

**Authors:** Ewelina Piktel, Katarzyna Pogoda, Maciej Roman, Katarzyna Niemirowicz, Grażyna Tokajuk, Marta Wróblewska, Beata Szynaka, Wojciech M. Kwiatek, Paul B. Savage, Robert Bucki

**Affiliations:** 1 Department of Microbiological and Nanobiomedical Engineering, Medical University of Bialystok, Bialystok, Poland; 2 Institute of Nuclear Physics, Polish Academy of Sciences, PL31342 Krakow, Poland; 3 Department of Periodontal and Oral Mucosa Diseases, Medical University of Bialystok, Bialystok, Poland; 4 Department of Dental Microbiology, Medical University of Warsaw, Warsaw, Poland; 5 Department of Microbiology, Central Clinical Hospital in Warsaw, Warsaw, Poland; 6 Department of Histology and Embryology, Medical University of Bialystok, Bialystok, Poland; 7 Department of Chemistry and Biochemistry, Brigham Young University, Provo, USA

## Abstract

Spore-forming bacteria are a class of microorganisms that possess the ability to survive in extreme environmental conditions. Morphological features of spores assure their resistance to stress factors such as high temperature, radiation, disinfectants, and drying. Consequently, spore elimination in industrial and medical environments is very challenging. Ceragenins are a new class of cationic lipids characterized by a broad spectrum of bactericidal activity resulting from amphipathic nature and membrane-permeabilizing properties. To assess the impact of ceragenin CSA-13 on spores formed by *Bacillus subtilis* (ATCC 6051), we performed the series of experiments confirming that amphipathic and membrane-permeabilizing properties of CSA-13 are sufficient to disrupt the structure of *B. subtilis* spores resulting in decreased viability. Raman spectroscopy analysis provided evidence that upon CSA-13 treatment the number of CaDPA-positive spores was clearly diminished. As a consequence, a loss of impermeability of the inner membranes of spores, accompanied by a decrease in spore resistance and killing take place. In addition to their broad antimicrobial spectrum, ceragenins possess great potential for development as new sporicidal agents.

Sporulation processes induced by limited nutrients characterize some bacteria, such as *Bacillus* and *Clostridium* species. It is well established that spore formation results in generation of metabolically dormant and environmentally resistant cells with abilities to survive high temperature, UV and gamma radiation, treatment with most antibiotics and extreme environmental conditions^1^,^2^,^3^. Moreover, spores are even 10- to 50- fold more resistant to the lethal effects of high vacuum, UV radiation and desiccation compared to vegetative cells^4^, making their eradication challenging for medical or industrial purposes. Spore-forming bacteria frequently occur in food products, and they are responsible for food spoilage and food-borne diseases and constitute an emerging problem in food production^5^. Importantly, dormant spores of some bacteria, mainly *Clostridium* species, are an infectious and transmissible form of the microbe. The persistence of spores, their germination, and their outgrowth into the vegetative form is responsible for a re-emergence of *Clostridium difficile* infections, even after long-term antibiotic treatment^6^,^7^.

Most research on sporicidal agents has focused on physical processes and application of reactive chemical compounds, many of which are toxic to humans, which be only applicable in industrial settings (e.g., sterilization with moist heat, UV radiation or hydrogen peroxide)^8^,^9^. Accordingly, the number of studies on antimicrobial agents effective in the treatment of infections caused by spore-forming bacteria is still very limited^10^. However, considering the pathogenicity of spore formers, the search for new sporicidal agents, appropriate for medical use is of significant interest.

Multiple factors are responsible for the resistance of *Bacillus* species spores. These include lower core water content and high mineralization of spore cores, relative impermeability of spore membranes, the spore core’s high level of dipicolinic acid (DPA) and its associated divalent cations (mostly CaDPA), the protection of spore DNA against wet heat damage by its saturation with α/β-type small, acid-soluble proteins (SASPs), the presence of UV-absorbing pigments located in the spore’s outer layers and DNA damage repair mechanisms during spore germination and outgrowth^8^,^11^,^12^. A major component in spore resistance is the unique multilayered structure of spores that differ from that of growing vegetative cell. This structure includes thick outer proteinaceous spore coats, outer membranes, cortexes, inner membranes and spore cores. Considering such complex spore structures, membrane-permeabilizing factors are attractive candidates as a novel sporicidal agents. An example of such agents is ceragenin CSA-13, a bile acid derivative designed to mimic the mechanism of action of endogenous antimicrobial peptides. A growing number of reports describing the broad-spectrum antimicrobial activity of antimicrobial peptides and ceragenins, primarily ceragenin CSA-13, leads to the hypothesis that ceragenins could possess anti-spore activity^13^,^14^,^15^,^16^,^17^,^18^,^19^,^20^. To date, it has been demonstrated that CSA-13 preferentially binds negatively charged phospholipids and causes alterations in the architecture of membranes (local loss of membrane organization, polarization and transport control)^21^. Membrane disruption and depolymerization occur as the consequence of the membrane activity of CSA-13, which can be enhanced via covalent immobilization onto magnetic nanoparticles surface^22^.

To date, there are several reports describing the potential ability of antibiotics to suppress sporulation processes^23^. However, therapies using classical antibiotics or synergistic activity of compounds with different mechanisms of action are often associated with high toxicity and low selectivity against spores. The complex structure of spores, in particular their inner membranes, must be considered to understand spore resistance to some chemicals and lytic agents^3^. Recognizing the membrane activity of CSA-13, we investigated whether permeabilizing properties of CSA-13 are sufficient to impair the inner membrane integrity of bacterial spores.

Although *B. subtilis* is not the major pathogen responsible for lethal infections in humans, it can serve as a model of spore formers suitable for studies of spore susceptibility. In this study, we assessed the sporicidal activity of ceragenin CSA-13 against *Bacillus subtilis* ATCC 6051, an undomesticated ancestor of the widely used *B. subtilis* strain 168^24^. We report that CSA-13 interacts with the spore structure which results in (i) alteration of spore coat integrity, (ii) increase in the permeability of spore membranes and (iii) loss of CaDPA. Combined, these effects decrease the resistance of spores to external factors and lead to decreased spore survival.

## Results

### CSA-13 exerts antibacterial activity against the vegetative form of B. subtilis and inhibits germination of spores

**Figure 1:**
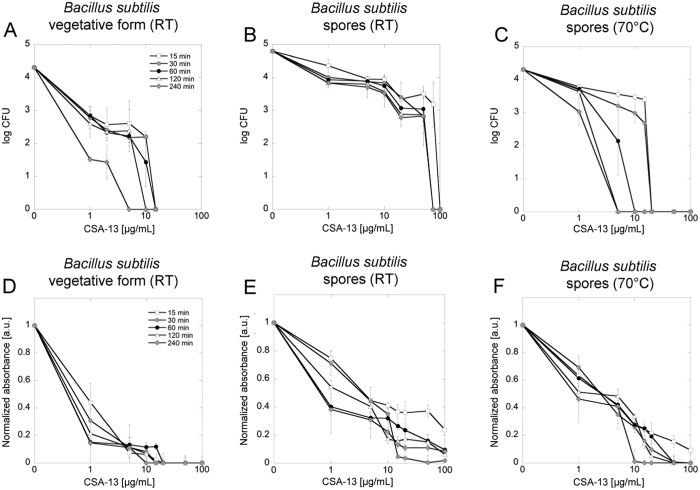
Decreased survival of CSA-13-treated *B. subtilis* spores. Decline in the survival of *B. subtilis* culture after incubation with various concentrations of CSA-13 (panels A–C) evaluated using a killing assay method. Level of metabolic activity observed in ceragenin-treated spore samples exposed to appropriate environmental conditions when compared to untreated control (panels D–F) assessed using the MTT assay. Samples were incubated at RT (panels A, D for vegetative form and panels B, E for spore form, respectively) and at 70 °C (panels C, F) for 15 (white squares), 30 (grey circles), 60 (black circles), 120 (white triangles) and 240 (grey diamonds) minutes. Data from experiments performed in triplicate are shown.

In the first phase of the study, the antibacterial activity of CSA-13 against *B. subtilis*, in vegetative form and in spore form, was assessed. As shown in Fig. 1A, CSA-13 exerted high antibacterial activity against the vegetative form of *B. subtilis* at concentrations ranging from 1 to 15 μg/mL. To assess whether the time of incubation significantly affected CSA-13 killing properties, incubation was extended to 4 h. Interestingly, the impact of incubation time was minimal; all vegetative cells were killed at a CSA-13 concentration of 15 μg/mL when incubation time reached 240 min. Simultaneously, incubation of a spore suspension at room temperature with CSA-13 at 75 μg/mL concentration resulted in total inhibition of their growth potential when the incubation time was equal or longer then 30 minutes (Fig. 1B). To assess the impact of higher temperature on spore viability, we performed additional incubations of spore suspensions with CSA-13 at 70 °C (Fig. 1C). It was confirmed that elevated temperature significantly intensifies the activity of CSA-13. In contrast to samples treated at room temperature, time- and dose-dependent effects during incubation in higher temperature were observed. Incubation lasting 15 min was sufficient to inhibit the growth of spores at a CSA-13 concentration of 20 μg/mL. Extension of incubation time to 120 and 240 minutes allowed the killing of bacterial spores at 5 μg/mL of CSA-13.

The antibacterial effects of CSA-13 (Fig. 1A–C) were confirmed using the tetrazolium salt MTT assay as a measure of viability (Fig. 1D–F). Dormant spores of *Bacillus* species exhibit low metabolism due to poor enzyme activity in the spore core and produce low levels of compounds such as NADH or ATP. However, the reactivation of spores and their return to vegetative metabolism, when the environmental conditions are suitable, is usually possible^25^. The incubation of non-treated spores in LB broth at 37 °C resulted in reactivation of metabolic activity confirmed by increasing formazan reduction and subsequent rise in an absorbance value (data not shown). Considering this fact, an additional test, allowing for detection of any metabolic activity in CSA-13-treated samples, was performed. According to collected data, in samples treated with corresponding doses of CSA-13, almost no metabolic activity was present. Vegetative cells showed no detectable metabolism when subjected to incubation with CSA-13 at 20 μg/mL, which indicates strong antimicrobial activity of this agent against vegetative form of bacteria (Fig. 1D). Importantly, only a small fraction of spores was able to resume proper metabolic activity after exposure to favorable environmental conditions – both when incubated at RT and elevated temperatures (Fig. 1E and F).

### CSA-13 interacts with spore structures

**Figure 2:**
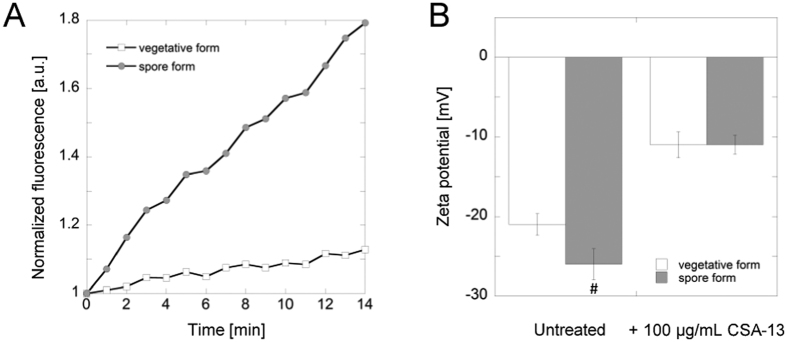
Affinity of FITC-labeled CSA-13 to vegetative and spore form of *B. subtilis* (panel A). Results from one representative of four experiments are shown. Changes in zeta potential values of ceragenin-treated vegetative and spore of *B. subtilis* (panel B). #Indicates statistical significance (P < 0.05) when compared to vegetative form of bacteria.

A number of studies have shown that ceragenins, including CSA-13, exert antimicrobial activity due to their amphipathic nature, which provides their membrane-permeabilizing properties upon insertion into bilayer lipid structures^26^,^27^,^28^. Consequently, CSA-13 membrane activity might explain its interference with the germination of *B. subtilis* spores indicated by results shown in Fig. 1. This observation was additionally confirmed by measurement of optical density at 600 nm (OD_600_). The dose-dependent changes of OD_600_ resulting from CSA-13 addition were observed (data not shown). Release of rhodamine 6G from spores treated with CSA-13 was also performed as an additional measure of its interference with spore structure. Spores incubation with CSA-13 at 20 μg/mL resulted in dye release, which strongly suggests an alteration in the charge density on the surface of the spore. Notably, this effect was significantly higher than that noted for vancomycin (data not shown). However, conditions of the experiment did not allow for a precise localization of rhodamine-spore binding, thus determination from which layer of spore structure the dye is being released is unknown and complicated by unspecific staining of spores with fluorescent dyes^29^. An additional assay employing FITC-labeled CSA-13 confirmed that CSA-13 exerts high affinity for the external layers of *B. subtilis* spores. Interestingly, CSA-13 possessed greater affinity for the spores than for the vegetative form of bacteria (Fig. 2A). To investigate the cause of this effect, the zeta-potential of *B. subtilis* in vegetative and spore forms was measured (Fig. 2B). It was confirmed that spores possessed more negative surface charge (−26 mV) than vegetative cells (−21 mV), which was the most likely cause of the differences in interactions with positively charged molecules such as CSA-13. Additionally, incubation of samples with 100 μg/mL CSA-13 led to a decrease in the absolute value of the observed zeta-potential (−11 mV).

### CSA-13 interrupts the integrity of the spore membrane causing changes in spore core morphology

**Figure 3:**
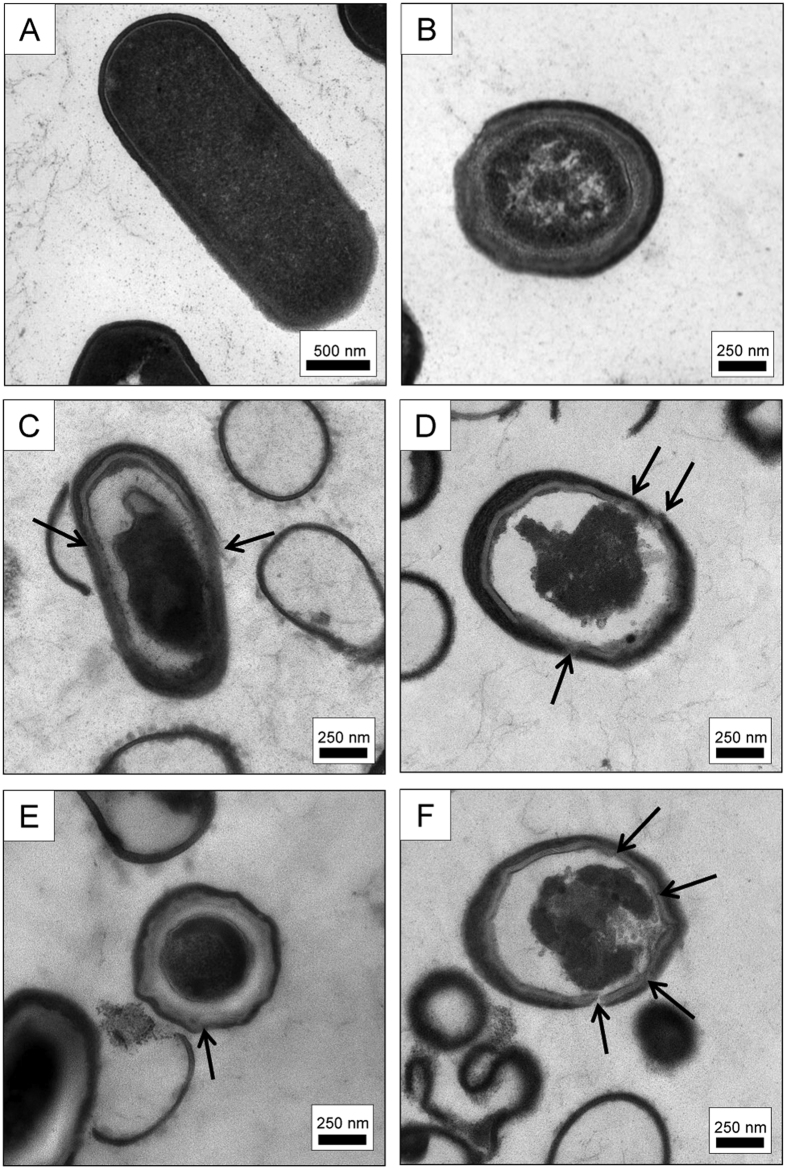
TEM micrographs of untreated vegetative (panel A) and spore form of *B. subtilis* (panel B). Disruption of spore membrane architecture after treatment with 50 μg/mL of CSA-13 at RT (panel C) and at 70 °C (panel D) and 200 μg/mL of CSA-13 at RT (panel E) and at 70 °C (panel F). Arrows indicates changes in membrane structure upon treatment with CSA-13.

TEM microscopy was performed to visualize the morphology of *B. subtilis* vegetative cells and spores upon exposure to CSA-13. Micrographs clearly illustrate that CSA-13 affects spore structure (Fig. 3). Both local and extensive changes in the integrity of external layers of spore led to alternations within spore core, which suggests that CSA-13 not only affects the structure of the spore coat but also increases the permeability of the outer and inner spore membranes. In particular, the ability of CSA-13 to by-pass the impermeability of spore’s inner membrane is considered as vital for the action of this agent, considering that maintained integrity of this layer plays a crucial role in the development of spore resistance to many chemicals^30^.

### CSA-13 induces the release of CaDPA from antibiotic-treated spores

**Figure 4:**
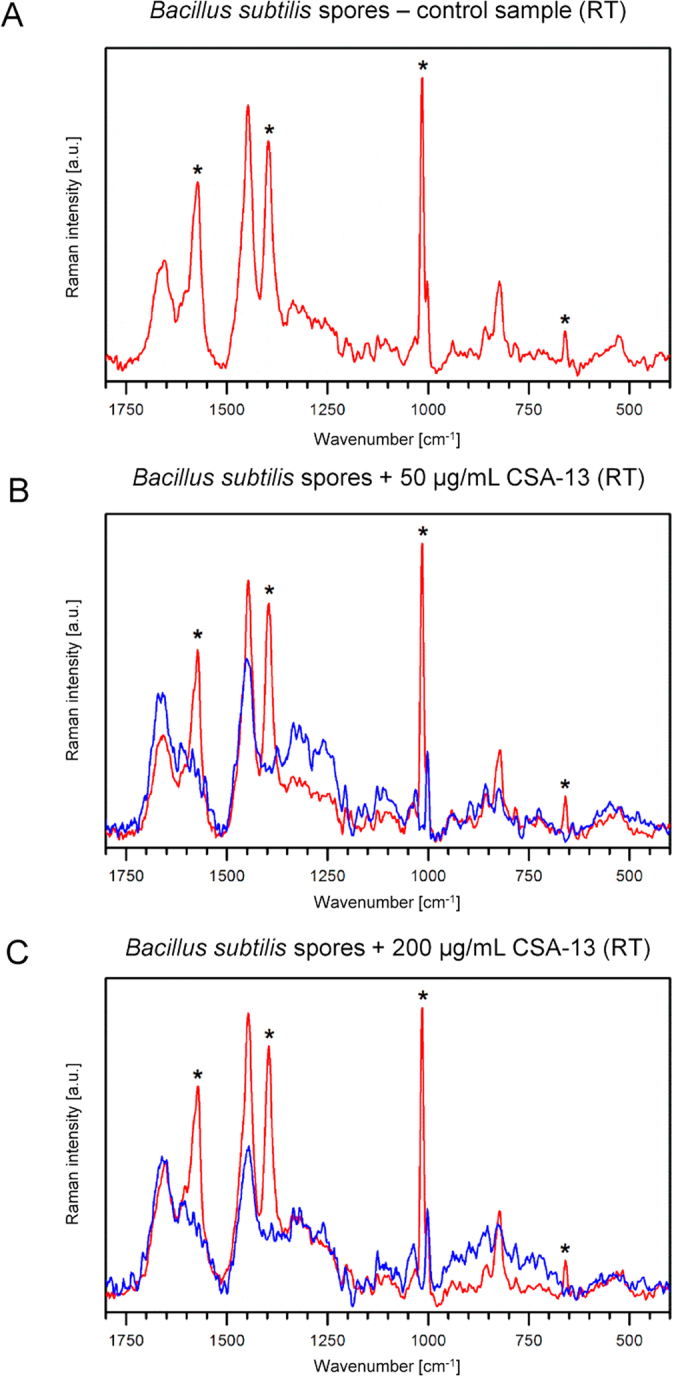
Raman spectra collected from *B. subtilis* spores treated with CSA-13 at RT. Panel A - control sample, panel B - 50 μg/mL of CSA-13, panel C - 200 μg/mL of CSA-13. Asterisks denote CaDPA bands well separated from bands of spore biocomponents. Blue color indicates spectra from CaDPA negative spores, while red color indicates spectra from CaDPA positive spores. All spores from control sample contained CaDPA (red spectra). Treatment with 50 μg/ml and 200 μg/ml of CSA-13 resulted in loss of CaDPA in 70% and and 45% of measured spores, respectively. Spectra were normalized to the 1002 cm^−1^ band. If the sample contained CaDPA positive and negative spores both spectra (blue and red) are presented.

**Figure 5:**
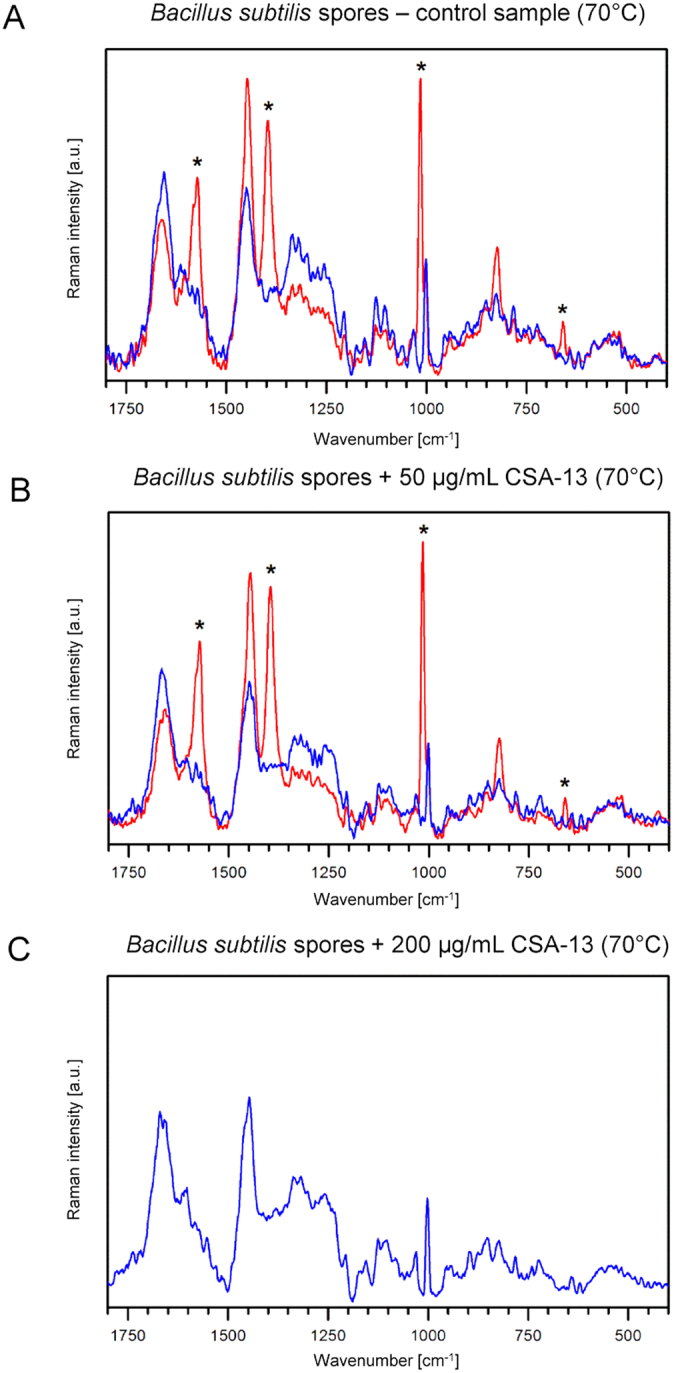
Raman spectra collected from *B. subtilis* spores treated with CSA-13 at 70 °C. Panel A - control sample, panel B - 50 μg/mL of CSA-13, panel C - 200 μg/mL of CSA-13. Asterisks denote CaDPA bands well separated from bands of spore biocomponents. Blue color indicates spectra from CaDPA negative spores, while red color indicates spectra from CaDPA positive spores. 90% of spores from control sample contained CaDPA. Treatment with 50 μg/ml and 200 μg/ml of CSA-13 resulted in loss of CaDPA in 95% and 100% of measured spores, respectively. Spectra were normalized to the 1002 cm^−1^ band. If the sample contained CaDPA positive and negative spores both spectra (blue and red) are presented.

DPA is considered to be one the key factors determining the resistance of *B. subtilis* spores to UV radiation and desiccation and as one of the molecules involved in the protection of DNA from damage^30^,^31^. Additionally, the release of this molecule occurs during killing of spores by wet heat and is preceded by an increase in inner membrane permeability^8^. Confocal Raman spectroscopy was used for analysis of changes in the chemical composition of spores upon treatment with CSA-13 (Fig. 4 and 5). DPA forms a complex with divalent ions (mostly Ca^2+^) and this complex exhibits characteristic bands in Raman spectra. The presence of these bands in spectra obtained from a single spore can be interpreted as high-CaDPA content cell (CaDPA positive), while their absence as low or undetectable CaDPA content cell (CaDPA negative). Following this approach, CSA-13 treated and untreated spores underwent spectroscopic evaluation. Based on Raman spectra, it was shown that incubation of spores with the ceragenin at room temperature resulted in a significant reduction of the number of CaDPA positive cells, while all untreated cells contained this complex. Treatment with 200 μg/ml of CSA-13 resulted in loss of CaDPA in 45% of measured spores. When treatment was conducted at 70 °C, the percentage of CaDPA negative spores was greater compared to sample treated at room temperature (Fig. 5). Treatment with 50 μg/ml of CSA-13 resulted in loss of CaDPA in 95% of measured spores, while treatment with 200 μg/ml of CSA-13 resulted in loss of CaDPA in all measured spores (no CaDPA positive spores were found). This effect agrees with observed increase of CSA-13 sporicidal activity at 70 °C (Fig. 1C and F).

## Discussion

Bacterial spores formed in sporulation processes are extremely resistant to physical sterilization, antimicrobials and antibiotics^1^,^2^,^3^. To date, a number of factors involved in the development of resistance of spores were recognized. Among them, the structure of spore plays a crucial role in the protection of cells against stressful environmental conditions. It is established that the spore coat is engaged in the resistance of spores against some chemicals and lytic enzymes degrading the spore cortex, but does not alter the resistance of cells against heat, radiation and select chemical decontaminants^2^. The outer membrane is not considered an important protecting barrier as well since its removal does not result in considerable alternations in spore resistance^2^,^32^. In contrast, the inner membrane of spores represents a strong permeability barrier, significantly defending internal structures of spores, mainly DNA, and playing a vital role in the development of chemical resistance^33^. Given the above, agents with the ability to by-pass permeability barriers of spores are promising in the treatment of this form of bacteria.

Membrane-permeabilizing properties of ceragenin CSA-13 described in a number of reports^13^,^14^,^21^ encouraged investigation of whether ceragenin-induced alterations in the organization of spore’s biological membranes might be sufficient to kill the spores of *B. subtilis* or considerably affect their germination and ability to outgrowth in appropriate environmental conditions. To date, it has been confirmed that similar mechanisms of sporicidal activity were described for oxidizing agents and acid solutions. It was reported that peroxynitrite and acids appear to kill spores by damaging the spore’s external layers, in particular, the inner membrane resulting in spore death^34^. On the other hand, treatment of spores with hydrogen peroxide causes dysfunction of spore germination^35^. However, these agents are not suitable to use in many medical treatments. Additionally, enzymes present in the spore coat, mainly superoxide dismutase, might detoxify some oxidizing agents before they penetrate into the deeper parts of the spore, which significantly reduced their usefulness^36^.

Our data indicate that the viability of vegetative cells and the ability of spores to germinate and restore outgrowth is strongly reduced after CSA-13 treatment. A substantial limitation of killing assays and the MTT test performed in the first stage of the study is the fact that it is not possible to state whether CSA-13 treated spores are indeed killed. Considering the nature of spore-forming microorganisms, including *Bacillus* spp. it is possible that CSA-13-treated spores are superdormant or unable to germinate under normal environmental conditions as a result of inactivation of some component of the spore’s germination apparatus. In 2002, it was demonstrated that viability of *B. subtilis* spores treated with strong alkali can be restored by germination with exogenous lysozyme, despite the fact that after plating of spores’ samples on rich media they seem to be dead^37^. However, while the exact mechanism of CSA-13 action was not definitely determined in this stage of the research, we can indicate that the CSA-13 treated spores lost the colony-forming ability and cannot take proper metabolism action upon exposure to this agent, as proven by optical density measurements combined with MTT assay. This also suggests that lack of metabolism detection in CSA-13-treated samples results from ceragenin-caused membrane disruption followed by loss of redox potential and proton motive force. This observation remains in agreement with previous studies showing that the OD_600_ of spore suspensions falls during spore germination due to alterations in the spore’s core refractive index upon water uptake and swelling of the core^38^. We hypothesized that this phenomenon is conditioned by the interaction of CSA-13 with spore’s external layers and to confirm this assumption we performed rhodamine 6G-release assay from stained spore cells (data not shown). In general, spores are difficult to stain with reagents that easily stain vegetative forms of bacteria. A fact that dye can be bound by almost all structures surrounding the spore core, including the outer proteinaceous spore coat, the thick peptidoglycan cortex and outer and inner membrane, is also an additional impediment in the determination from which layer dye is released upon CSA-13 treatment^35^,^37^. Nevertheless, our observed release of rhodamine 6G indicates that CSA-13 disturbs the integrity of the multilayered structure of spores. The affinity of FITC-labeled CSA-13 to spore layers was also confirmed by fluorescence-based measurements (Fig. 2A).

Interestingly, it was revealed that CSA-13 interacts stronger with spores than with vegetative forms of bacteria. To understand the source of such phenomena we carried out zeta potential measurement for both vegetative and spore form of *Bacillus*. Considering the cationic nature of the CSA-13, we suggested that differences in surface charge of spores and vegetative cells might result in altered interaction of this compound with the biological membrane of these cells. Our measurements confirmed these predictions, showing that bacterial spores possess more negatively charged cell surface than vegetative cells, which is most likely associated with the local charge of the proteins that comprise the outer layers of the spore. Additionally, studies demonstrated by Kosh *et al*. and Kemper *et al*. suggest as well that physiologically active *B. subtilis* cells possess less negatively charged surface than inactive cells, which is conditioned by protonation of bacterial cell wall during growth and lower pH near the surface^39^,^40^. Noteworthy, incubation of samples with CSA-13 led to a decrease of the negative charge of vegetative cells and spores, which indicates its interaction with the biological membrane of cells.

A significant result obtained from our study is the release of CaDPA from CSA-13 treated spore samples. As presented in Fig. 4 and 5, almost all untreated spores contain high levels of this complex, while ceragenin-treated samples are characterized by its significant loss, reaching our threshold of detection. This effect can be strengthened by exposure of spores to elevated temperatures. It is established that the high level of DPA (∼20% of core dry weight) considerably improves the resistance of spore to external stress factors; however, its role in this phenomenon is still unclear. It is well-known that DPA occurs in the spore core as 1:1 chelate with divalent cations, predominantly Ca^2+ ^31 and its presence in spore allows for decrease of core water content by replacing some core water with DPA, which promote the resistance of spores to hydrogen peroxide, formaldehyde, and the iodine-based disinfectants^41^. Additionally, DPA is involved in the protection of spore DNA from damage caused by dry heat or desiccation^42^. Importantly, the level of residual spore core water is sufficient to maintain the antimicrobial activity of CSA-13, since spore core is not completely dehydrated^41^. To date, it has been assumed that increased levels of DPA are correlated with wet heat resistance of spores^41^. However, studies performed by Balassa *et al*.^43^ and Hanson *et al*.^44^ indicate that the presence of DPA is not the only factor involved in heat-resistance of spores since isolation of thermo-resistant DPA-less spores is also possible. Despite these reports, it is assumed that the protection of spore core proteins from denaturation and inactivation by wet heat promotes wet heat resistance^41^.

**Figure 6:**
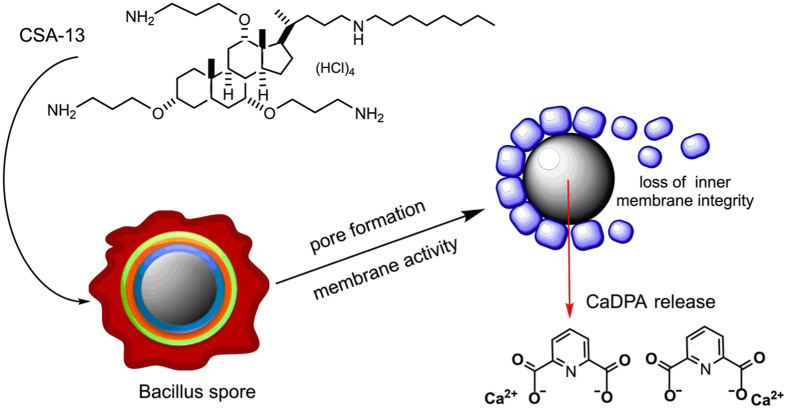
Proposed mechanism of ceragenin CSA-13 sporicidal activity.

In our study, we demonstrated that increasing incubation temperature significantly affected the killing properties of CSA-13. As presented in the Fig. 1C and F (viability assay) and Fig. 5 (Raman measurements) the sporicidal activity of ceragenin was greatly strengthened when the spores were exposed to higher temperatures in aqueous environment. We were unable to determine the precise mechanism of this phenomenon, however, our observations are with the agreement with reports demonstrated by Russell *et al*. indicating the improvement of sporicidal activity of chemicals at elevated temperatures^45^. We hypothesize that this effect could be associated with alternations in core water content, which would be consistent with reported studies demonstrating a decrease in spore resistance to sterilizing agents due to greater spore core hydration^9^,^46^. Considering that the loss of spore DPA is followed by replacement of this compound by water^8^, we suggest that alterations in core hydration are associated with the CSA-13-induced release of CaDPA from spore core as the result of membrane-permeabilizing properties of this agent (Fig. 6). Research performed by Coleman *et al*. confirmed our conclusion. Based on observations noted by this research team, it was determined that the major event during treatment of spores with moist heat is the release of DPA from the spore core, and this process results from alterations in permeability of spore barriers, probably the spore inner membrane, which allows for increased accessibility of the spore core to exogenous factors. Importantly, this process involves a simultaneous increase in spore hydration^8^. Given the above, we hypothesized that a similar mechanism of action against spores was involved in the observed activities of CSA-13. So we assumed that both treatment methods decrease the viability of spore by comparable mechanisms. It is possible that the augmented killing properties of CSA-13 at higher temperatures in aqueous environments result from CaDPA release, which is followed by eradication of the DPA-depleted spores and increased sensitivity to sporicidal, chemical agents.

On the other hand, DPA release from the spore core is an event that occurs during germination of *B. subtilis* spores due to the activation of germinant receptors, and it is established that DPA release activates cortex degradation and activates the second stage of this process^47^. Considering this fact, it can be proposed that the mechanism of action of CSA-13 involves triggering of germination process followed by eradication of germinated spores and vegetative cells, which are more sensitive to external factors than spores alone. However, our observations including the low level of outgrowth of CSA-13-treated spores in appropriate nutrient conditions and lack of water influx observed in the optical density measurements are not consistent with this possibility.

In conclusion, we suggest that further studies evaluating the anti-spore properties of CSA-13 and other ceragenins are justified. Our study clearly indicates that ceragenin CSA-13, apart from its pleiotropic antimicrobial properties^48^,^49^,^50^,^51^, possesses great potential for development of a new sporicidal agent. However, additional studies are needed to determine the exact mechanism of action involved in the activity of this compound against *B. subtilis* spores.

## Material and Methods

### Spore preparation and purification

To prepare *Bacillus subtilis* ATCC 6051 spores, bacteria were grown on LB agar plates (tryptone 10.0 g/L, yeast extract 5.0 g/L, sodium chloride 10.0 g/L, agar 15.0 g/L) without antibiotics at 37 °C for 72 h. Bacteria were harvested, re-suspended in ice-cold phosphate buffered saline (PBS) solution and purified by triple centrifugation (12 000× g, 10 min, 4 °C). The spore suspension was heat treated (75 °C, 15 min) to remove remaining vegetative cells, cooled, stored in water at 4 °C and protected from light until analysis^52^,^53^,^54^. Previous studies confirmed that heat activation with subsequent cooling does not significantly affect the properties of spores and their resistance to external factors^55^. All spores used in this work were free of vegetative cells (>98%), as determined by staining cells using Schaeffer and Fulton Spore Stain Kit (Sigma-Aldrich, St Louis, MO, USA) (data not shown). Additionally, to confirm the formation of spores during preparation, purified samples were investigated using transmission electron microscopy (TEM, Tecnai G2 X-TWIN, FEI, USA). TEM micrographs confirmed the presence of *B. subtilis* spores with characteristic spore-exclusive structures (Fig. 3B).

### The viability of Bacillus subtilis spores and vegetative cells

Viability assay was performed according to the protocol presented by Ghosh and Setlow^38^. Spore suspensions in PBS were brought to 10^8 ^CFU/mL and incubated with various concentrations of CSA-13. To evaluate the effect of treatment time on the killing properties of the antibiotic, incubation was performed for 15, 30, 60, 120 and 240 min, respectively. To investigate the effect of temperature on sporicidal properties, incubation at room temperature (RT) and at 70 °C was performed. After incubation, the plates were transferred to ice and suspensions were diluted 10- to 1000-fold in PBS. Then, 10 μL aliquots were spotted on LB agar plates for overnight culture at 37 °C and CFUs were determined. Cell survival, after exposure to the tested agent, was expressed as percent of control.

As an additional confirmation of results, the presence of a metabolically active fraction of cells was investigated using MTT assay. Briefly, after incubation of spores with various concentrations of CSA-13 in a non-growing medium (PBS), 20 μL of MTT solution (thiazolyl blue tetrazolium bromide, Sigma-Aldrich, St Louis, MO, USA, 5 mg/mL) and 100 μL of LB medium broth were added. Incubation at 37 °C was continued for 8 h. Medium was removed, and 100 μL of dimethyl sulfoxide solution (DMSO) was added to dissolve the MTT precipitate. Cells were allowed to stand at RT for 10 min with shaking. Absorbance values were detected at a wavelength of 570 nm using a microplate spectrophotometer. Absorbance values obtained in control spores cultures (without a tested agent) were taken as 100%. As positive controls, 1 M HCl (for spore treatment at RT) and 70% ethanol (for spore treatment at 70 °C) were employed. The average of all the experiments was presented in comparison to the level of metabolic activity detected in non-treated *B. subtilis* suspension. Incubation of both vegetative cells and spores of *B. subtilis* was performed for 15, 30, 60, 120 and 240 min.

### Measurement of optical density

To assess germination processes in antibiotic-treated samples, a spectrophotometric method was employed. Briefly, isolated spores were incubated at RT for 1 h in PBS with concentrations of CSA-13 ranging from 10 to 100 μg/mL. Then 100 μL of LB broth was added, and optical densities at 600 nm (OD_600_) were monitored, using a microplate reader (Synergy H1, BioTek, VT, USA), for 60 min.

### CSA-13-induced release of rhodamine 6G from spore structures.

The release of rhodamine 6G absorbed on the surface of *Bacillus* spores was evaluated using the Maesaki method^56^ with minor modifications. To stain cells with rhodamine 6G, the dye was added to a final concentration of 10 μM for 10 min. Non-absorbed dye was removed by centrifugation for 2 min at 10 000× g. Next, stained cells were washed and CSA-13 at 20 μg/mL was added. After incubation for 10, 30, 60 and 120 min, supernatant was collected by centrifugation and absorption at 527 nm was measured. The total concentration of rhodamine 6G released from the surface of spores was calculated using a standard concentration curve. The level of antibiotic-induced release was presented as the difference between the concentration of rhodamine 6G released from treated spores and control samples.

### Measurement of zeta potential and affinity of antibiotic

To assess the affinity/binding of CSA-13 for the outer bacterial membrane, CSA-13 was labeled with fluorescein isothiocyanate (FITC) and added to the suspensions of vegetative cell and spores, to a final concentration of 20 μg/mL. The affinity of CSA-13 to cell membranes was assessed using fluorimetric measurement (Synergy H1, BioTek, VT, USA) with excitation/emission wavelengths of 298/534 nm recorded for 15 min. To evaluate whether affinity of CSA-13 is influenced by the surface electrical properties of cells, zeta potentials of vegetative bacteria and spore suspensions were assessed using Zetasizer Nano ZS (Malvern Instruments, United Kingdom). Bacterial and spore cultures were brought to OD_600_ ~ 0.1 in PBS buffer (pH = 7). To evaluate the effect of the ceragenin on zeta potential value, CSA-13 was added at the concentration of 100 μg/mL to the spore suspension, incubated for 15 minutes and transferred to a cuvette. Measurements were conducted at 25 °C.

### Transmission electron microscopy TEM

To visualize alterations in morphology and membrane permeability of treated spores, a suspension of spores (OD_600_~0.5) in distilled water was treated with CSA-13 at concentrations of 50 μg/mL and 200 μg/mL and incubated for 1 h at RT and 70 °C. After incubation, cells were centrifuged (8000 rpm, 5 minutes) and samples were fixed in a mixture of 2.5% glutaraldehyde and 2% paraformaldehyde in 0.1 M cacodylate buffer (CB) at pH 7.0 for 1 h at 4 °C, and taken up into agar blocks. Then samples were washed in CB at 4 °C (1 h) and post-fixed in 1% osmium tetroxide in CB for 1 h at 4 °C and next dehydrated through a graded series of ethanol and embedded in Glycid ether 100 (Epon 812). Ultrathin sections were contrasted with uranyl acetate and lead citrate and mounted on nickel grids and evaluated in a transmission electron microscope OPTON 900.

### Confocal Raman spectroscopy

For Raman spectroscopy analyses, spores of *B. subtilis* were suspended in 100 μL of distilled water and treated with CSA-13 (50 μg/mL and 200 μg/mL) for 1 h at RT and 70 °C. Samples were transferred to polished calcium fluoride (CaF_2_) optical windows (Crystran, United Kingdom) and dried at 60 °C. Raman spectra were recorded using a Renishaw InVia Raman spectrometer equipped with an optical confocal microscope, an air-cooled laser emitting at 532 nm, and an CCD detector thermoelectrically cooled to −70 °C. A dry Leica N PLAN EPI (100x, NA 0.85) objective was used. The power of the laser at the sample position was ca. 1.5 mW. A sum of 20 scans with integration time of 20 s and a resolution of 0.5 cm^−1^ was collected. The spectrometer was calibrated using the Raman scattering line generated by an internal silicon plate. A laser spot (diameter of *ca.* 760 nm) was focused on a single spore and then the measurement was performed. All spectra were smoothed and baseline corrected. Results from one representative experiment are provided.

## Additional Information

**How to cite this article:** Piktel, E. *et al*. Sporicidal activity of ceragenin CSA-13 against *Bacillus subtilis.* *Sci. Rep.* **7**, 44452; doi: 10.1038/srep44452 (2017).

**Publisher's note:** Springer Nature remains neutral with regard to jurisdictional claims in published maps and institutional affiliations.

## References

[b1] Russell A. D. Bacterial spores and chemical sporicidal agents. Clin Microbiol Rev 3, 99–119 (1990). 2187595 10.1128/cmr.3.2.99 PMC358146

[b2] Nicholson W. L., Munakata N., Horneck G., Melosh H. J. & Setlow P. Resistance of *Bacillus* endospores to extreme terrestrial and extraterrestrial environments. Microbiol Mol Biol Rev 64, 548–572 (2000). 10974126 10.1128/mmbr.64.3.548-572.2000 PMC99004

[b3] Driks A. *Bacillus subtilis* spore coat. Microbiol Mol Biol Rev 63, 1–20 (1999). 10066829 10.1128/mmbr.63.1.1-20.1999 PMC98955

[b4] Nicholson W. L., Schuerger A. C. & Setlow P. The solar UV environment and bacterial spore UV resistance: considerations for Earth-to-Mars transport by natural processes and human spaceflight. Mutat Res 571, 249–264, doi: 10.1016/j.mrfmmm.2004.10.012 (2005). 15748651

[b5] Lücking G., Stoeckel M., Atamer Z., Hinrichs J. & Ehling-Schulz M. Characterization of aerobic spore-forming bacteria associated with industrial dairy processing environments and product spoilage. Int J Food Microbiol 166, 270–279, doi: 10.1016/j.ijfoodmicro.2013.07.004 (2013). 23973839

[b6] Depestel D. D. & Aronoff D. M. Epidemiology of *Clostridium difficile* infection. J Pharm Pract 26, 464–475, doi: 10.1177/0897190013499521 (2013). 24064435 PMC4128635

[b7] Paredes-Sabja D., Shen A. & Sorg J. A. *Clostridium difficile* spore biology: sporulation, germination, and spore structural proteins. Trends Microbiol 22, 406–416, doi: 10.1016/j.tim.2014.04.003 (2014). 24814671 PMC4098856

[b8] Coleman W. H., Chen D., Li Y. Q., Cowan A. E. & Setlow P. How moist heat kills spores of *Bacillus subtilis*. J Bacteriol 189, 8458–8466, doi: 10.1128/JB.01242-07 (2007). 17890306 PMC2168948

[b9] Popham D. L., Sengupta S. & Setlow P. Heat, hydrogen peroxide, and UV resistance of *Bacillus subtilis* spores with increased core water content and with or without major DNA-binding proteins. Appl Environ Microbiol 61, 3633–3638 (1995). 7486999 10.1128/aem.61.10.3633-3638.1995 PMC167661

[b10] Bouillaut L. . Effects of Surotomycin on *Clostridium difficile* Viability and Toxin Production *In Vitro*. Antimicrob Agents Chemother 59, 4199–4205, doi: 10.1128/AAC.00275-15 (2015). 25941230 PMC4468702

[b11] Fairhead H., Setlow B. & Setlow P. Prevention of DNA damage in spores and *in vitro* by small, acid-soluble proteins from *Bacillus* species. J Bacteriol 175, 1367–1374 (1993). 8444799 10.1128/jb.175.5.1367-1374.1993 PMC193223

[b12] Magge A. . Role of dipicolinic acid in the germination, stability, and viability of spores of *Bacillus subtilis*. J Bacteriol 190, 4798–4807, doi: 10.1128/JB.00477-08 (2008). 18469099 PMC2446992

[b13] Leszczynska K. . Antibacterial activity of the human host defence peptide LL-37 and selected synthetic cationic lipids against bacteria associated with oral and upper respiratory tract infections. J Antimicrob Chemother 68, 610–618, doi: 10.1093/jac/dks434 (2013). 23134677 PMC3566669

[b14] Leszczyńska K. . Potential of ceragenin CSA-13 and its mixture with pluronic F-127 as treatment of topical bacterial infections. J Appl Microbiol 110, 229–238, doi: 10.1111/j.1365-2672.2010.04874.x (2011). 20961363 PMC3386848

[b15] Byfield F. J. . Cathelicidin LL-37 increases lung epithelial cell stiffness, decreases transepithelial permeability, and prevents epithelial invasion by *Pseudomonas aeruginosa*. J Immunol 187, 6402–6409, doi: 10.4049/jimmunol.1102185 (2011). 22095714

[b16] Wnorowska U. . Bactericidal activity of cathelicidin LL-37 and select cationic lipids against the hypervirulent *P. aeruginosa* strain LESB58. Antimicrob Agents Chemother, doi: 10.1128/AAC.00421-15 (2015). PMC4468669 25870055

[b17] Surel U., Niemirowicz K., Marzec M., Savage P. B. & Bucki R. Ceragenins – a new weapon to fight multidrug resistant bacterial infections. Studia Medyczne 30, 207–213 (2014).

[b18] Wnorowska U. . Extracellular DNA as an essential component and therapeutic target of microbial biofilm. Studia Medyczne 31, 132–138 (2015).

[b19] Leszczyńska K. . Bactericidal activities of the cationic steroid CSA-13 and the cathelicidin peptide LL-37 against *Helicobacter pylori* in simulated gastric juice. BMC Microbiol 9, 187, doi: 10.1186/1471-2180-9-187 (2009). 19728885 PMC2748089

[b20] Bucki R. & Janmey P. A. Interaction of the gelsolin-derived antibacterial PBP 10 peptide with lipid bilayers and cell membranes. Antimicrob Agents Chemother 50, 2932–2940, doi: 10.1128/AAC.00134-06 (2006). 16940084 PMC1563552

[b21] Howell M. D. . Ceragenins: a class of antiviral compounds to treat orthopox infections. J Invest Dermatol 129, 2668–2675, doi: 10.1038/jid.2009.120 (2009). 19516269 PMC8609773

[b22] Niemirowicz K. . Bactericidal activity and biocompatibility of ceragenin-coated magnetic nanoparticles. J Nanobiotechnology 13, 32, doi: 10.1186/s12951-015-0093-5 (2015). 25929281 PMC4458011

[b23] Aldape M. J., Heeney D. D., Bryant A. E. & Stevens D. L. Tigecycline suppresses toxin A and B production and sporulation in *Clostridium difficile*. J Antimicrob Chemother 70, 153–159, doi: 10.1093/jac/dku325 (2015). 25151204 PMC4267498

[b24] Zeigler D. R. . The origins of 168, W23, and other *Bacillus subtilis* legacy strains. J Bacteriol 190, 6983–6995, doi: 10.1128/JB.00722-08 (2008). 18723616 PMC2580678

[b25] Setlow P. Mechanisms which contribute to the long-term survival of spores of *Bacillus* species. Soc Appl Bacteriol Symp Ser 23, 49S–60S (1994). 8047910 10.1111/j.1365-2672.1994.tb04357.x

[b26] Savage P. B., Li C., Taotafa U., Ding B. & Guan Q. Antibacterial properties of cationic steroid antibiotics. FEMS Microbiol Lett 217, 1–7 (2002). 12445638 10.1111/j.1574-6968.2002.tb11448.x

[b27] Lai X. Z. . Ceragenins: cholic acid-based mimics of antimicrobial peptides. Acc Chem Res 41, 1233–1240, doi: 10.1021/ar700270t (2008). 18616297

[b28] Saha S., Savage P. B. & Bal M. Enhancement of the efficacy of erythromycin in multiple antibiotic-resistant gram-negative bacterial pathogens. J Appl Microbiol 105, 822–828, doi: 10.1111/j.1365-2672.2008.03820.x (2008). 18452533

[b29] Magge A., Setlow B., Cowan A. E. & Setlow P. Analysis of dye binding by and membrane potential in spores of *Bacillus* species. J Appl Microbiol 106, 814–824, doi: 10.1111/j.1365-2672.2008.04048.x (2009). 19187156 PMC2661013

[b30] Setlow P. Spores of *Bacillus subtilis*: their resistance to and killing by radiation, heat and chemicals. J Appl Microbiol 101, 514–525, doi: 10.1111/j.1365-2672.2005.02736.x (2006). 16907802

[b31] Setlow B., Atluri S., Kitchel R., Koziol-Dube K. & Setlow P. Role of dipicolinic acid in resistance and stability of spores of *Bacillus subtilis* with or without DNA-protective alpha/beta-type small acid-soluble proteins. J Bacteriol 188, 3740–3747, doi: 10.1128/JB.00212-06 (2006). 16707666 PMC1482921

[b32] Setlow B., McGinnis K. A., Ragkousi K. & Setlow P. Effects of major spore-specific DNA binding proteins on *Bacillus subtilis* sporulation and spore properties. J Bacteriol 182, 6906–6912 (2000). 11092849 10.1128/jb.182.24.6906-6912.2000 PMC94814

[b33] Cortezzo D. E. & Setlow P. Analysis of factors that influence the sensitivity of spores of *Bacillus subtilis* to DNA damaging chemicals. J Appl Microbiol 98, 606–617, doi: 10.1111/j.1365-2672.2004.02495.x (2005). 15715863

[b34] Genest P. C., Setlow B., Melly E. & Setlow P. Killing of spores of *Bacillus subtilis* by peroxynitrite appears to be caused by membrane damage. Microbiology 148, 307–314 (2002). 11782523 10.1099/00221287-148-1-307

[b35] Melly E., Cowan A. E. & Setlow P. Studies on the mechanism of killing of *Bacillus subtilis* spores by hydrogen peroxide. J Appl Microbiol 93, 316–325 (2002). 12147081 10.1046/j.1365-2672.2002.01687.x

[b36] Henriques A. O., Melsen L. R. & Moran C. P. Involvement of superoxide dismutase in spore coat assembly in *Bacillus subtilis*. J Bacteriol 180, 2285–2291 (1998). 9573176 10.1128/jb.180.9.2285-2291.1998 PMC107166

[b37] Setlow B. . Mechanisms of killing spores of *Bacillus subtilis* by acid, alkali and ethanol. J Appl Microbiol 92, 362–375 (2002). 11849366 10.1046/j.1365-2672.2002.01540.x

[b38] Ghosh S. & Setlow P. Isolation and characterization of superdormant spores of *Bacillus* species. J Bacteriol 191, 1787–1797, doi: 10.1128/JB.01668-08 (2009). 19136594 PMC2648361

[b39] Koch A. L. The pH in the neighborhood of membranes generating a protonmotive force. J Theor Biol 120, 73–84 (1986). 3018380 10.1016/s0022-5193(86)80018-2

[b40] Kemper M. A., Urrutia M. M., Beveridge T. J., Koch A. L. & Doyle R. J. Proton motive force may regulate cell wall-associated enzymes of *Bacillus subtilis*. J Bacteriol 175, 5690–5696 (1993). 8396121 10.1128/jb.175.17.5690-5696.1993 PMC206628

[b41] Paidhungat M., Setlow B., Driks A. & Setlow P. Characterization of spores of *Bacillus subtilis* which lack dipicolinic acid. J Bacteriol 182, 5505–5512 (2000). 10986255 10.1128/jb.182.19.5505-5512.2000 PMC110995

[b42] del Carmen Huesca Espitia L., Caley C., Bagyan I. & Setlow P. Base-change mutations induced by various treatments of *Bacillus subtilis* spores with and without DNA protective small, acid-soluble spore proteins. Mutat Res 503, 77–84 (2002). 12052506 10.1016/s0027-5107(02)00093-3

[b43] Balassa G., Milhaud P., Raulet E., Silva M. T. & Sousa J. C. A *Bacillus subtilis* mutant requiring dipicolinic acid for the development of heat-resistant spores. J Gen Microbiol 110, 365–379, doi: 10.1099/00221287-110-2-365 (1979). 108357

[b44] Hanson R. S., Curry M. V., Garner J. V. & Halvorson H. O. Mutants of *Bacillus cereus* strain T that produce thermoresistant spores lacking dipicolinate and have low levels of calcium. Can J Microbiol 18, 1139–1143 (1972). 4627143 10.1139/m72-175

[b45] Russell A. D. & Loosemore M. Effect of phenol on *Bacillus subtilis* spores at elevated temperatures. Appl Microbiol 12, 403–406 (1964). 14215968 10.1128/am.12.5.403-406.1964 PMC1058144

[b46] Paidhungat M. & Setlow P. Role of ger proteins in nutrient and nonnutrient triggering of spore germination in *Bacillus subtilis*. J Bacteriol 182, 2513–2519 (2000). 10762253 10.1128/jb.182.9.2513-2519.2000 PMC111315

[b47] Setlow P. Germination of spores of *Bacillus* species: what we know and do not know. J Bacteriol 196, 1297–1305, doi: 10.1128/JB.01455-13 (2014). 24488313 PMC3993344

[b48] Niemirowicz K. . Magnetic nanoparticles enhance the anticancer activity of cathelicidin LL-37 peptide against colon cancer cells. Int J Nanomedicine 10, 3843–3853, doi: 10.2147/IJN.S76104 (2015). 26082634 PMC4461127

[b49] Bozkurt-Guzel C., Savage P. B. & Gerceker A. A. *In vitro* activities of the novel ceragenin CSA-13, alone or in combination with colistin, tobramycin, and ciprofloxacin, against *Pseudomonas aeruginosa* strains isolated from cystic fibrosis patients. Chemotherapy 57, 505–510, doi: 10.1159/000335588 (2011). 22286090

[b50] Kuroda K. . Ceragenin CSA-13 induces cell cycle arrest and antiproliferative effects in wild-type and p53 null mutant HCT116 colon cancer cells. Anti-cancer drugs 24, 826–834, doi: 10.1097/CAD.0b013e3283634dd0 (2013). 23817390

[b51] Piktel E. . The Role of Cathelicidin LL-37 in Cancer Development. Arch Immunol Ther Exp (Warsz) 64, 33–46, doi: 10.1007/s00005-015-0359-5 (2016). 26395996 PMC4713713

[b52] Swiecicka I., Fiedoruk K. & Bednarz G. The occurrence and properties of *Bacillus thuringiensis* isolated from free-living animals. Lett Appl Microbiol 34, 194–198 (2002). 11874541 10.1046/j.1472-765x.2002.01070.x

[b53] Vaerewijck M. J. . Occurrence of *Bacillus sporothermodurans* and other aerobic spore-forming species in feed concentrate for dairy cattle. J Appl Microbiol 91, 1074–1084 (2001). 11851816 10.1046/j.1365-2672.2001.01477.x

[b54] Xi-Yang. Studies on the impact of probiotic bacteria on enteric microbial diversity and immune response. University of Wollongong (2006).

[b55] Zhang P., Setlow P. & Li Y. Characterization of single heat-activated *Bacillus* spores using laser tweezers Raman spectroscopy. Opt Express 17, 16480–16491 (2009). 19770863 10.1364/OE.17.016480

[b56] Maesaki S., Marichal P., Vanden Bossche H., Sanglard D. & Kohno S. Rhodamine 6G efflux for the detection of CDR1-overexpressing azole-resistant *Candida albicans* strains. The Journal of antimicrobial chemotherapy 44, 27–31 (1999). 10459807 10.1093/jac/44.1.27

